# Walnut Male Flowers (*Juglans regia* L.) as a Functional Addition to Wheat Bread

**DOI:** 10.3390/foods11243988

**Published:** 2022-12-09

**Authors:** Karolina Pycia, Agata Maria Pawłowska, Joanna Kaszuba, Natalia Żurek

**Affiliations:** Department of Food Technology and Human Nutrition, Institute of Food Technology and Nutrition, University of Rzeszow, 4 Zelwerowicza Street, 35-601 Rzeszow, Poland

**Keywords:** *J. regia*, flowers, wheat bread, functional food, antioxidant activity, total polyphenol

## Abstract

The aim of this study is to assess the effect of adding dried and ground walnut male flowers to wheat flour on selected physicochemical and antioxidant properties of the obtained wheat bread. The control sample was bread without the addition of walnut male flowers. In the tests, the addition of flowers was used in the amounts of 0.5%, 1.0%, 1.5%, 2.0% and 2.5% of the weight of the flour used in the control sample. Bread dough was prepared according to the direct method with the use of yeast. The parameters of the baking process of the control bread and of the test bread were calculated. The selected parameters determining the quality of the obtained bread, including the loaf volume, the specific volume, the crumb porosity, the crumb texture and the color were measured. The antioxidant potential and total polyphenol content were tested both in the dough and in the bread. The test results were analyzed statistically. It was found that the partial replacement of wheat flour with walnut male flowers resulted in a significant reduction in the loaves’ volume and in the darkening of both the color of the dough and the crumb. The value of the L* parameter decreased with the increasing addition of walnut flowers. The lowest values of this parameter were found to be 53.87 (crust) and 39.94 (crumb) in the sample with 2.5% addition. The average volume of the loaves ranged from 565 cm^3^ (0.5%) to 675 cm^3^ (control). The use of the additive in the amount of 2.5% caused significant changes in the values of most of the examined parameters of the bread crumb texture. The addition of walnut male flowers to the flour had a significant effect on the antioxidant potential and the total polyphenol content of the tested doughs and breads. In the case of bread, the total polyphenol content value ranged from 96.90 mg GAE/g dw to 208.08 mg GAE/g dw. Similarly, the antioxidant potential increased with increasing walnut male flower supplementation. The antioxidant potential determined by the ABTS and FRAP methods ranged from 1.42 mmol TE/g dw to 2.02 mmol TE/g dw and from 0.08 mmol TE/g dw to 0.33 mmol TE/g dw, respectively. The obtained research results prove the application potential of walnut flowers in the design of food, with beneficial pro-health properties, and are an example of the use of plant by-products in the process of food enrichment.

## 1. Introduction

Contemporary consumers are looking for food that, apart from a high nutritional value, also provides bioactive ingredients helpful in diet and prophylaxis [1]. It has long been proven that the right diet plays an important role in the prevention of chronic diseases. At the same time, a diet rich in natural antioxidants reduces the risk of free radical diseases. Following this, the efforts of food technologists and scientists focus on the development of food products into which raw materials are intentionally incorporated, to give the finished products functional properties [2]. This is possible thanks to the use of ingredients that are a natural source, e.g., polyphenols with a different chemical structure. The natural sources of polyphenols are plant materials, which differ in terms of their content and profile [3]. The latest research results confirm that valuable bioactive ingredients are also present in the inedible parts of many plants or by-products from their processing. Phenolic compounds, in addition to their widely documented antioxidant activity, exhibit antimicrobial and antifungal activity, and have a significant impact on the sensory quality of food products [4]. Among the plant materials that are a rich and valuable source of polyphenols are walnuts, i.e., the seeds of the tree of the same name and by-products from their processing, as well as other parts of the plant (flowers, leaves).

The walnut is a tree that belongs botanically to the Juglandaceae family. The region it comes from is Central Asia, in the western Himalayan chain and the Kyrgyzstan region. This species reached Europe before Roman times and later spread to the countries of America and North Africa [5,6]. The appearance of the kernels resembles the human brain; therefore walnut seeds are considered a rich, natural source of substances supporting the functioning of the central nervous system. They have been shown to contain antioxidant phytochemicals such as ellagic acid, melatonin, vitamin C, carotenoids, polyphenols and more. In addition to their antioxidant effect, they also delay the aging processes, cancer and inflammation [7]. Apart from the seeds, also valuable in terms of nutrients are green walnuts, and the shells, bark and leaves, which are therefore used in the cosmetic and pharmaceutical industries [8]. Kernels are, in addition, a dietary food that is used to treat diabetes and inflammation. The infusion of the leaves has antidiarrheal, anthelmintic and depurative effects. The bark has an astringent, diuretic, bactericidal activity and many other effects [9]. Apart from those mentioned above, walnut flowers are also rich in biologically active substances [9,10]. The latest research has shown that the content of polyphenols in the extract of male walnut flowers was as high as 4369.73 mg/100 g dw. Twenty-four compounds belonging to the group of flavonoids, phenolic acids and juglanosides have been identified in the polyphenol profile. A strong correlation was also demonstrated between the content of polyphenols and the antioxidant capacity and cytotoxic activity of the examined extract. According to the cited authors, walnut male flowers are a natural and valuable source of compounds with antioxidant, antibacterial and chemopreventive properties that can be used in the pharmaceutical and food industries [10]. In the case of the food industry, *J. regia* flowers can be used to enrich food with bioactive substances, which has not yet been described in the literature. A well-known and easily available product that can be enriched is wheat bread. Bread and other bakery products play an important role in human nutrition, mainly as a source of energy. The proportion and quality of raw materials, as well as the technology of making dough and baking, affect the quality and nutritional value of bread. Bread produced from extract flours is still more often chosen by consumers, while in the aleurone layer of wheat grain and bran, there are many valuable bioactive substances lost during milling [2,11]. Thus, there is a need to enrich the bread to improve its nutritional value and health benefits, which, as a consequence of these activities, will allow new products to be offered to consumers. Bread is often used as a carrier of bioactive substances present in plant raw materials used to fortify bakery products, mainly due to the ease of dosing additives and the quick assessment of the results of such a recipe or technological innovations. Pycia and Ivanisova [12] and Pycia and Kapusta [13] showed that wheat bread enriched with dry walnuts, hazelnuts and nuts with various degrees of maturity, respectively, was characterized by a higher antioxidant potential compared to the control samples. It is difficult to find information in the scientific literature on the possibility of using *J. regia* male flowers to enrich bread. Therefore, this constitutes an innovative aspect of the presented research and will contribute by indicating the application possibilities of walnut male flowers and to the development of an interesting food product. Thus, the aim of the study was to assess the potential of using walnut flowers to enrich wheat bread and to assess its quality.

## 2. Materials and Methods

### 2.1. Materials and Reagent

Sodium carbonate, ABTS (2,20-azino-bis-(3-ethylbenzothiazoline-6-sulfonic acid), TPTZ (2,4,6-tris-2,4,6-tripyridyl-2-triazine), iron (III) chloride, Folin–Ciocalteu reagent, methanol, acetic acid (glacial), hydrochloric acid, Trolox (6-hydroxy-2,5,7,8-tetramethylchromane-2-carboxylic acid) and gallic acid were used for analyses. All reagents were purchased from Sigma-Aldrich (Darmstadt, Germany) and Chempur (Piekary Śląskie, Poland).

#### 2.1.1. Plant Materials

Walnut male flowers (WF) (*J. regia* L.) were harvested in May 2022 from flowering walnut trees growing in the Subcarpathian region (Poland). The flowers were dried using a freeze dryer (ALPHA 1–2 LD plus Martin Christ Gefriertrocknungsanlagen GmbH, Osterode am Harz, Germany), ground in a laboratory mill (MPM, Milanówek, Poland) and used for analysis. Wheat flour type 650 (Polskie Młyny, Warszawa, Poland) with 52.8% water absorption determined by farinograph (Farinograph-E, Brabender, Duisburg, Germany) [14] and 13.6% moisture content was used to bake the bread. The wheat flour contained 27.5% of wet gluten, which was characterized by a gluten index deformation value of 6 mm [15], and its falling number was 384 s [16]. Baker’s yeast (LALLEMAND Polska, Józefów, Poland), table salt (o’Sole, Września, Poland) and potable tap water were also used.

#### 2.1.2. Preparation of Bread

The control dough was prepared using the direct method [12,13,17,18]. The dough was made of wheat flour (known to be water-absorbing), baker’s yeast (3% by weight of flour), salt (1% by weight of flour) and tap water at 30 °C in an amount that allows a dough consistency of 350 FU (farinographic units) to be obtained, which was calculated on the basis of the known water absorption of flour [17]. In the tested systems, wheat flour was partially replaced by dried, ground walnut flowers at the level of 0.5%, 1.0%, 1.5%, 2.0% and 2.5% of the flour weight of the control bread dough (0.0, control), and the obtained test breads were coded A, B, C, D, E, respectively, according to the increasing proportion of the additive. A laboratory mixer R4 (Mesko-AGD, Skarżysko-Kamienna, Poland) was used to combine the ingredients and mix the dough. The dough was fermented for 60 min at 30 °C in a fermentation chamber (Sveba Dahlen, Fristad, Sweden), with a breakthrough after 30 min (time 2 min, manually). After the first fermentation, the dough was weighed and cut into 250 g portions, which were hand formed into the shape of a ball. The dough pieces were placed in greased baking tins and post-fermented until optimal growth was obtained. Once the fermentation process was completed, the tins with the dough pieces were placed in the baking chamber of the Classic electric oven (Sveba Dahlen, Sweden) and baked at 230 °C for 30 min. After baking, the bread was weighed (hot bread) and allowed to cool on metal grids. The quality of the baked bread was tested 24 h after baking. Preparation of the dough, bread baking and quality tests were performed in triplicate.

### 2.2. Methods

#### 2.2.1. Parameters of the Baking Process and Bread Quality

Laboratory baking parameters, such as dough yield (%), bread yield (%), oven loss (%), total oven loss (%) were determined [17]. The loaves’ volume (cm^3^) was determined by the loose millet seed displacement method according to AACC Standard 10-05.01 [19]. The specific volume of bread (cm^3^/g) was also calculated [20]. The crumb porosity was assessed by comparing digital photos of the crumb taken with a 13 MPix camera (Samsung, Suwon, Republic of Korea) and the photos of the crumb from Dallmann tables presenting the scale with assigned porosity coefficients [21].

#### 2.2.2. The Color of the Dough and Bread

Measurements of color parameters were made for the dough samples before the fermentation process and for the crust and crumb of the cooled bread using a spectrophotometer (HunterLab, Reston, VA, USA). The conditions for measuring the parameters of the CIE L*a*b* system were as follows, measurement in reflection: measuring geometry d/8°, illuminate D65, range 400–700 nm, detector deflected 10°, measurement gap 25 mm [12]. The parameter L* was marked, denoting the brightness of the sample. The parameters +a*, −a*, +b*, −b* denote the proportion of red, green, yellow and blue colors, respectively. The absolute color difference between the control sample and the test sample (∆E) was calculated [22]:

$$\Delta E=\sqrt{{\left(\Delta L\right)}^{2}+{\left(\Delta a\right)}^{2}+{\left(\Delta b\right)}^{2}}$$

Measurements were made in triplicate for each type of dough and bread.

#### 2.2.3. Bread Crumb Texture Analysis

The analysis of the parameters of the crumb texture was performed using the EZ-LX texture analyzer (Shimadzu, Kyoto, Japan) with the dedicated Trapezium X Texture Pl software. A sample of crumb from the central part of the loaf, in the shape of a cube a = 30 mm, was compressed twice along its axis with a stainless-steel disc with a diameter of d = 25 mm at a speed of v = 50 mm·s^−1^ until 50% deformation of the initial height was obtained. On the basis of the obtained results, the following parameters of the texture of the bread crumb were determined: hardness (N), elasticity (-), cohesiveness (-), gumminess (N), chewiness (N) and resilience (-). Measurements of the texture parameters of each type of baked bread were performed in four replications.

#### 2.2.4. Antioxidant Potential and Total Polyphenol Content in Dough and Bread

##### Sample Preparation

For the analysis of total polyphenol content and antioxidant potential, methanol extracts of doughs and breads were used. The doughs and breads were frozen, lyophilized and ground. The powdered material was mixed in a 1:5 ratio with methanol (50%, *v*/*v*) and extracted using ultrasound (Sonic 10 ultrasonic bath, Polsonic, Poland) at 30 °C for 30 min. The mixture was centrifuged, the solution decanted and the extraction procedure was repeated again with methanol (80%, *v*/*v*). All extracts were prepared on the day of chemical analysis.

##### Total Polyphenols Content

The total content of polyphenols was determined by the Folin–Ciocalteu method described by Gao et al. [23]. In brief, 0.1 mL of samples, 2 mL of distilled water, 0.2 mL of Folin–Ciocalteu reagent and 1 mL of sodium carbonate (20%, *w*/*v*) were transferred into spectrophotometric cuvettes, mixed and left for 1 h in a dark place. Then, the absorbance was measured at a wavelength of 765 nm using a UV–Vis spectrometer (Type UV2900, Hitachi, Japan). The results were expressed as gallic acid equivalent per g of dry weight (mg GAE/g dw).

##### Antioxidant Activity

ABTS (2,2’-azino-bis (3-ethylbenzothiazoline-6-sulfonic acid)) Radical Scavenging Assay.

The scavenging activity was carried out by a spectrophotometric method using the ABTS cation radical, according to Re et al. [24]. Three milliliters of ABTS solution was mixed with 0.03 mL of the sample. After 6 min, the absorbance was measured at a wavelength of 734 nm.

Ferric Reducing Antioxidant Power (FRAP).

The FRAP test was determined by a spectrophotometric method described by Benzie and Strain [25]. Three mL of FRAP solution were added to 0.5 mL of the sample. The absorbance was recorded at a wavelength of 593 nm after 10 min of reaction.

The values of the antioxidant activity of walnut extracts were expressed in mM Trolox Equivalent per 100 g dry weight (TE/100 g dw).

### 2.3. Statistical Analysis

The obtained test results were subjected to the one-way analysis of variance (ANOVA). The significance of differences between the mean values was assessed by Duncan’s test at the significance level *p* ≤ 0.05. Moreover, between the values of the parameters characterizing the dough and bread, Pearson’s linear correlation coefficients (*p* ≤ 0.05) were determined. Statistical analysis was performed using the Statistica 13.3 program (TIBCO Software Inc., Palo Alto, CA, USA).

## 3. Results and Discussion

### 3.1. Baking Process Parameters

The technological quality of wheat bread depends primarily on the baking value of the flour, but also on the type and amount of other ingredients used. The use of good-quality flour with the simultaneous use of ingredients to improve the nutritional or health-promoting value of the bread may contribute to the deterioration in the quality of the obtained bread, which is most often caused by the weakening of dough gluten [3,26]. Table 1 summarizes the parameters of the baking process of wheat bread enriched with WF. It was shown that the tested doughs did not differ significantly in terms of efficiency. In turn, the baking loss and the total baking loss generally decreased as the proportion of WF in the recipe increased. The lowest value of these parameters was found in bread with a 2% addition of WF. Gumul et al. [27] also noted a decrease in the values of these parameters due to the addition of apple pomace, which is a source of polyphenols. The authors reported a reduction in both the baking loss and the total baking loss, with the high content of the fiber fraction of the additive used. In turn, in the studies by Cacak-Pietrzak et al. [28], an increase in baking loss was noted due to the addition of dried cistus leaves. The yield of wheat bread with the addition of at least 1% WF differed significantly from both the control sample and breads with a lower share of the discussed additive (Table 1). The value of this parameter, in the case of bread with the addition of 2% WF, increased by 5% compared to the control sample. The studies of authors such as Cacak-Pietrzak et al. [29], regarding the addition of ground dried dandelion roots or onion husks to the production of bread [30], did not confirm a significant effect of these additives on the yield of wheat bread. In other studies [28], the authors noted a significant increase in the efficiency of the obtained bread with the addition of purge (3%, 4%, 5%). The bread efficiency depends on the amount of water absorbed by the flour, and according to the quoted authors, the increase in water absorption is due to the use of an additive with very low moisture content, which in turn increases the water absorption of the mixture used for baking bread.

The bread’s volume is the basic parameter that determines the quality of the bread, which significantly influences the consumer’s purchasing decision. This parameter also indirectly informs about crumb aeration, its porosity and texture [22]. The discussed research results showed that the addition of walnut flowers had a statistically significant effect on the reduction in the volume of wheat bread (Table 2), and the value of this parameter ranged from 675 cm^3^ (control) to 565 cm^3^ (E). Similarly, as the share of WF in the bread recipe increased, its specific volume decreased linearly (2.62–3.21 cm^3^/g) (Table 2). The performed statistical analysis showed a significant, strong linear correlation between the volume of loaves and the bread yield, specific volume, and dough color parameters L*, a* (r = −0.93, r = 0.99, r = 0.96; r = −0.88, respectively; *p* < 0.05). The enrichment of bread with various plant additives generally reduces the volume of the loaves due to the weakening of the gluten network and/or inhibition of the activity of baker’s yeast. This is confirmed by the results of research on enriching bread with, for example, sesame seeds [31], oilseeds [18], Moringa Oleifera leaf powder [20], walnuts and hazelnuts [12], nuts with various degrees of maturity [13], dried parsley leaves [22] or the purge [28] and many others. In the conducted research, wheat flour containing gliadin and glutenin was partially replaced with walnut flowers. Probably, the addition not only reduced the amount of gluten proteins in the system but also weakened the gluten structure. As a result, the weakened dough cannot retain the carbon dioxide produced in the fermentation process. According to Dziki et al. [22], a reduction in the volume of bread loaves generally increases the hardness of the crumb. Moreover, the reduction in the volume of bread loaves under the influence of WF may result from the high content of polyphenols [10], which show an antimicrobial effect. Probably, the polyphenols present in WF reduced the activity of yeast responsible for the fermentation of the dough and the production of carbon dioxide, the amount of which is directly related to the volume of the loaves. In earlier studies [13], a reduction in the volume of loaves of bread was noticed due to the addition of unripe walnuts and hazelnuts rich in polyphenols. Indeed, Żurek et al. [10] identified the presence of 24 bioactive compounds in walnut flowers. Nine of them constituted the flavonoid group, 12 compounds belonged to phenolic acid derivatives and 3 were considered to be juglanoside isomers. The cited authors also proved the antimicrobial potential of the WF extract towards Gram (+) and Gram (−) bacteria. Probably, this effect is also related to the decrease in the volume of the fortified bread. Czubaszek et al. [3] also observed a reduction in the volume of wheat bread loaves under the influence of the addition of plant extracts rich in polyphenols. Baiano et al. [32], enriching wheat bread with extracts from vegetable waste, also observed a reduction in the volume of loaves of bread. The cited authors explained this fact by the probable denaturation of gluten proteins under the influence of extracts from plant waste, which had a lower pH than water. Moreover, bioactive substances present in plant raw materials may form complexes between proteins/polysaccharides, and thus also influence the development of the dough [32,33]. According to Sivam et al. [34], such complexes are stabilized by several chemical bonds, such as hydrogen, covalent or ionic bonds between phenolic compounds and proteins. The porosity of the crumb of the tested bread with smaller proportions of additive (0.5 and 1.0%) was assessed as being worse compared to the control sample, while the greater proportion of the additive resulted in an improvement in the discussed parameter (Table 2). The best crumb porosity was found in bread with the highest (2.5%) share of walnut flowers. This is similar to previous studies by other authors who noted that the crumb porosity of wheat bread gradually increased with increasing herbal content from 1% to 5% [28].

### 3.2. Colour of Dough and Bread

Table 3 presents the results of the measurements of the color parameters of the dough with the addition of WF. Walnut flowers are a plant material, so they contain chlorophyll in their chemical composition, which will have the greatest influence on the color of the dough, crust and bread crumb. It was found that the brightness of the dough determined by the L* parameter values decreased statistically significantly with the increasing share of WF in the recipe. The L* value for the dough with the highest WF content was nearly 30% lower than the result of the control sample. Statistically significantly, also changed was the value of the a* parameter in relation to the control sample, i.e., the share of red color increased. In the case of the value of the b* parameter showing the share of yellow and blue colors, similarly to the a* parameter, it was shown that the value increased in the range from 16.37 (control) to 18.37 (E). Thus, along with the increasing share of WF in the recipe, the share of the yellow color increased. The value of the ∆E parameter indicates the possibility of perceiving the difference in the color of the compared objects made by the observer. The highest value of this parameter, calculated in relation to the control sample, was recorded in the tests of dough with 2.5% WF addition, and the value of ∆E increased statistically significantly along with the increase in the share of WF in the dough. The performed statistical analysis showed a significant, strong linear correlation between the L* parameter values and the dough yield, loaf volume and specific volume (r = −0.93, r = 0.96, r = 0.93, *p* < 0.05, respectively). The color of the dough associated with the addition of WF significantly influenced the color of the crust and the crumb of the bread (Table 3). Both the color of the crumb and the surface (crust) of the bread affect consumer acceptance and the decision to buy it.

**Figure 1:**
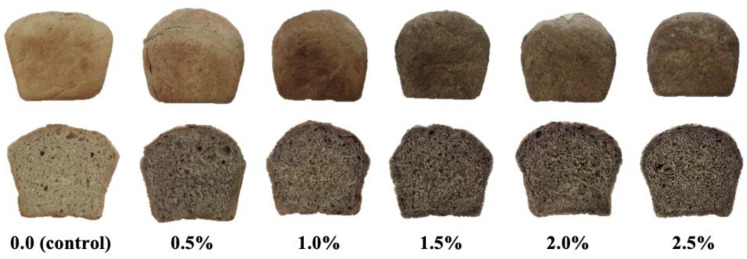
The appearance of the loaves and crumb of the tested wheat breads.

It was found that the addition of WF had a statistically significant effect on the color of both the crust and the crumb of bread enriched with WF. In both cases, the replacement of part of the flour with WF decreased the value of the L* parameter, which indicates the brightness. With increasing WF addition, a darker color of both the crust and the crumb of the bread, was observed. The value of the L* parameter in the tests of the crust and the crumb of the bread with 2.5% addition was lower by nearly 16% and 43%, respectively, in relation to the results of the lightness of the crust and the crumb of the control sample. The values of parameters a* and b* also changed. In the case of the crust in enriched breads, the values of these parameters were lower compared to the control sample, i.e., the share of red and yellow colors decreased. In turn, in the case of the bread crumb, the values of the a* parameter in WF breads were higher compared to the control sample, and the b* parameter was lower, i.e., the share of red color increased and the yellow color decreased. Pycia and Ivanisova [12] and Pycia and Kapusta [13] observed similar changes in the case of breads enriched with nuts with dry hazelnuts and walnuts as well as nuts with different degrees of maturity. In the case of the latter, the dominant dye, as with the walnut flowers used in the present research, was chlorophyll. The ∆E parameter informs about the noticeability of the difference in the color of products, visible even by an inexperienced observer. Some authors believe that the total color difference ∆E < 1 is not noticeable to the human eye, values in the range 1 < ∆E < 3 are the color differences noticeable to the experienced observer and ΔE > 3 is regarded as the threshold value for the obvious color difference of the compared objects [35]. According to the criterion established by the International Commission on Illumination (CIE), the total color difference ΔE between 0 and 2 is insignificant and unrecognizable by an experienced and skilled observer, while a total color difference greater than 5 is significant and recognizable even by an inexperienced observer [36]. Both in the case of the crust and the crumb, the enrichment of the bread with the addition of walnut flowers at the level of 0.5% resulted in a noticeable color difference compared to the control sample (Table 4). The value of this parameter increased statistically significantly with the increasing share of WF in the recipe. The color changes in the crumb of the bread enriched with a different addition of WF are shown in Figure 1. The color of food products can be shaped with the use of natural or synthetic dyes. The former are found in plant raw materials, which, when used as enriching additives, shape not only the color of the product but also its nutritional value or pro-health value [22]. As with the results from previous studies of enriching bread with dried parsley leaves [22] or purge [28], they caused a clear darkening of the bread crumb and an increase in the proportion of red in the crumb color. Czubaszek et al. [3] also observed changes in the color of the crumb of bread enriched with plant extracts. The direction of changes depended on the properties of the extract.

### 3.3. Textural Parameters of Bread Crumb

The texture of bakery products plays a key role in shaping their commercial quality and consumers’ acceptance. On one hand, enriching the bread with health-promoting substances increases the nutritional value of the bread, and on the other hand, it affects the rheological properties of the dough, the course of the fermentation process and the quality of the crumb in the finished product [37]. In the discussed studies (Table 4), it was found that the hardness of the bread crumb did not differ significantly statistically as the share of WF in the bread recipe increased in the range of 0.5–2.0% compared to the control sample. According to Dziki et al. [22], in general, the crumb hardness increases with the reduction in the bread volume, which was confirmed in the discussed studies only in the case of bread with the highest proportion of walnut flowers (Table 4). Moreover, Cacak-Pietrzak et al. [28] did not find a significant change in the hardness of bread with the addition of 1%, 2% and 3% purge compared to the control bread. The tested breads, the control and bread with the addition of WF (0.5 and 1.0%) did not differ in terms of crumb elasticity (Table 5). The value of this parameter decreased statistically significantly along with the increase in the share of WF in the recipe in the range of 1.0–2.5%. A slight effect of the share of WF on the cohesiveness of the bread crumb was observed, and it was not different from the value of this parameter obtained in the study of the crumb of the control sample. The highest chewiness of the crumb was found to be in the bread with 2.5% addition of walnut flowers, while the highest chewiness of the crumb was in the bread with 1.5% addition of walnut flowers (Table 5). There was no difference in the resilience of the crumb of the control bread and the breads with 0.5% and 1% WF addition, and the greater share of the additive resulted in a decrease in the value of this parameter in the other tested breads. Pycia and Kapusta [13] found an increase in the hardness, gumminess and chewiness of the crumb of bread enriched with dried unripe walnuts, and the increase in the value of these parameters was the greater the greater the share of walnuts was. It should be noted that, according to Pycia et al. [6], unripe walnuts are characterized by a very high content of bioactive compounds with a high antioxidant potential, which is likely to affect the fermentation process, the volume of loaves of bread and the nature of the crumb. It can be assumed that a similar phenomenon took place in the discussed research on breads with the addition of walnut male flowers. 

### 3.4. Antioxidant Activity and Total Phenolic Content in Dough and Bread

Enriching bread by replacing part of the flour with various herbs, spices and green parts of plants is a new and recognized food fortification trend. Roots, stems, leaves, seeds and flowers are a rich source of micro- and macroelements, dietary fiber, bioactive substances, phytochemicals and essential oils. Green parts of plants are the most valuable source of bioactive ingredients due to the oxygen produced in these parts by photosynthesis [2,38].

**Figure 2:**
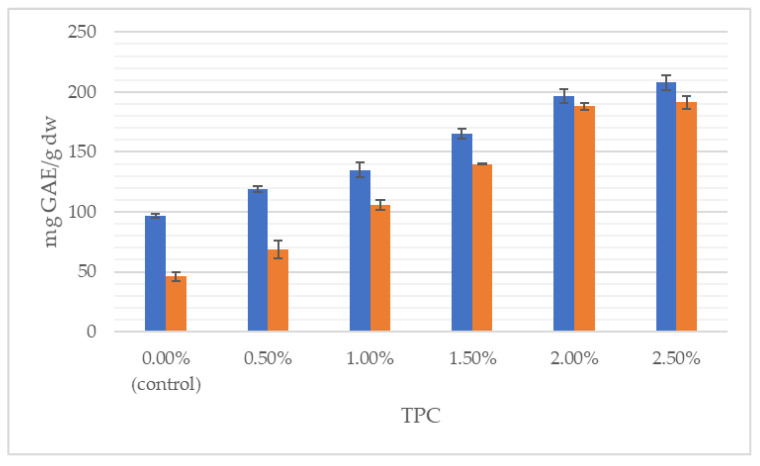
Total polyphenol content (TPC) of wheat dough (blue bars) and bread (orange bars) enriched with *J. regia* male flowers.

**Figure 3:**
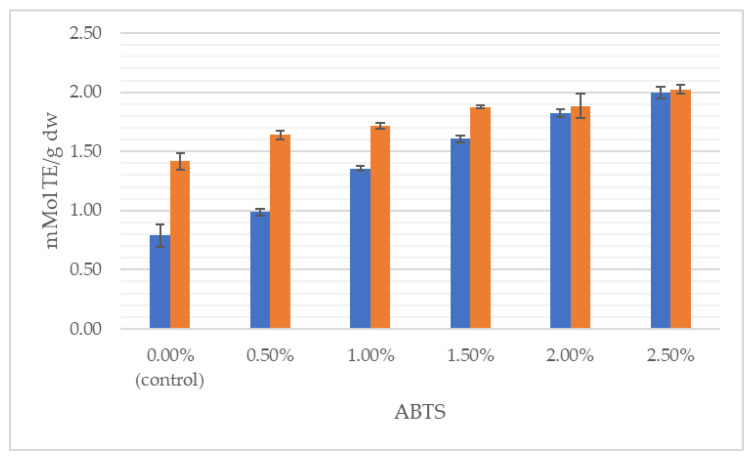
Antioxidant activity measured by ABTS method of wheat dough (blue bars) and bread (orange bars) enriched with *J. regia* male flowers.

**Figure 4:**
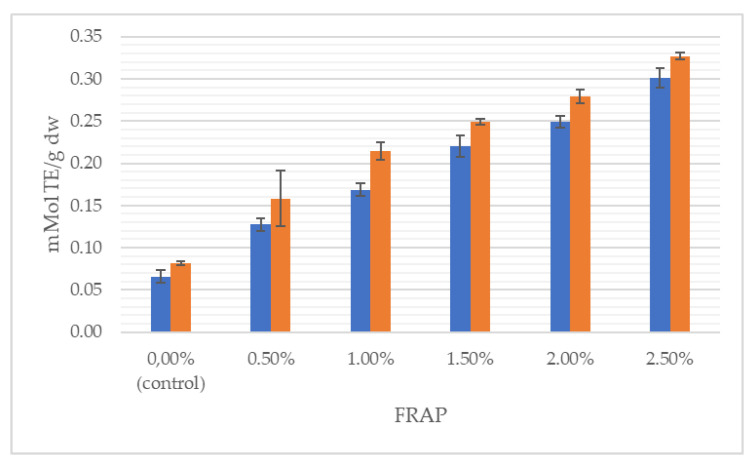
Antioxidant activity measured by FRAP method of wheat dough (blue bars) and bread (orange bars) enriched with *J. regia* male flowers.

Figure 2, Figure 3 and Figure 4 illustrate the average total polyphenol content and the antioxidant activity determined by ABTS and FRAP assays in doughs and breads enriched with *J. regia* male flowers, respectively. 

It can clearly be seen that the total content of polyphenolics grew with the increasing share of flowers, both in the dough and in the final product. With respect to the control, TPC was incremented by 115% in the wheat dough enriched with 2.5% of walnut flowers and by 316% in 2.5% fortified bread. Likewise, antioxidant properties progressively increased with augmentation from 0.5 to 2.5% of walnut flower powder. For instance, the addition of 2.5% of inflorescences lyophilizate caused antioxidant power in the dough and bread to be raised, respectively, by 363% and 299%, measured by the FRAP method. In the study of Dziki et al. [22], the addition of dried parsley leaves to wheat bread increased the content of phenols. The authors found a significant (*p* < 0.05) and positive linear relationship between the multiplicity of the additive and the TPC value. Bread extracts with a 2% share of the additive were characterized by about twice the content of phenolic compounds compared to the control bread, while in the discussed studies, the use of a 2.5% addition of walnut flowers resulted in a nearly 4-fold higher content of this group of compounds.

A similar effect on the TPC level and antioxidant potential was noted in the study of Cacak-Pietrzak et al. [29] on the enrichment of wheat bread with dried and ground dandelion roots.

The statistical analysis showed a correlation between the TPC of the dough and the performance of bread, loaf volume, specific volume, the L* parameter of the dough and ∆E of the dough (r = 0.92, r = −0.96, r = −0.96, r = −0.98; *p* < 0.05). Similarly, linear correlations were found between the L* crumb parameter and the bread yield, loaf volume, specific volume, L* dough parameter and ∆E of the dough (r = 0.89, r = −0.93, r = −0.93, r = −0.97; *p* < 0.05). The antioxidant potential of bread depends on the production conditions and the recipe composition. The process of making bread involves three stages, such as making dough, fermentation and baking [2]. During these stages, changes in the antioxidant potential of the dough are observed. As proven, the mixing process generally lowers the antioxidant potential, and during fermentation and baking, the antioxidant potential is recovered due to the hydrolysis of chemical bonds during fermentation and the release of antioxidants [2]. According to Han and Koh [39], baking bread allows for the recovery of lost antioxidants at an average level of 70–80%.

Few authors have shown that walnut male inflorescences are a valuable source of phenolic compounds [9,10,40,41,42,43]. As individual polyphenolic constituents, Muzzafer and Paul [9] revealed the presence of gallic acid, myricetin and quercetin derivatives. Pop et al. [40] identified several phenolic acids, catechin and quercetin glycosides with hyperoside, isoquercitrin and quercitrin being the predominant constituents. Similarly, Zhang et al. [41] determined a wide range of phenolic acids with ellagic acid being the main one. Chrzanowski et al. [42], instead, as the prevalent compound in the flowers, reported vanillic acid. Recently, Żurek et al. [10] found that walnut flowers contain compounds from the group of flavonoids, phenolic acids and juglanosides. In their study, the most abundant constituents were quercetin 3-*O*-glucoside, followed by quercetin diglucoside and 5-*O*-caffeoylquinic acid. The discrepancies in the phytochemical profile may vary by species, season, habitat or the ecological conditions of plants. However, in all of the cited studies a strong correlation between the content of polyphenols and the antioxidant capacity activity was observed.

Interestingly, the TPC in the bread decreased in relation to the TPC in the dough (Figure 2), while the antioxidant activity of bread increased after baking (Figure 3 and Figure 4). This phenomenon has already been observed [39]. Although polyphenols are sensitive to light, oxygen, temperature and pH variations, and are susceptible to storage degradation, melanoidins are formed through a Maillard type reaction during baking. It has been repetitively shown that melanoidins have strong antioxidant properties, thus, may contribute to the antioxidant power of baked bread [39,44]. The Maillard reaction products and phenol compounds are good at scavenging peroxyl and ABTS radicals; therefore, the antioxidant potential of bread may depend on the synergistic interaction of many factors, including, inter alia, thermal product degradation and the formed polyphenol–polysaccharide complexes [32,34,45]. Pycia and Ivanisowa [12] and Pycia and Kapusta [13] showed an increase in the antioxidant potential and total polyphenol content in breads enriched with dry walnuts and hazelnuts as well as walnuts and hazelnuts of various degrees of maturity. However, an increase in the antioxidant activity of wheat bread was noted in other studies on the use of different types of additives, i.e., leaves, e.g., parsley [22] and onion [46]; roots, e.g., dandelion [29]; fruit, e.g., sea buckthorn [47] and citrus albedo [48]; and flowers, e.g., hibiscus [49].

## 4. Conclusions

Walnut male flowers (WF) are an unconventional raw material with a high content of bioactive substances, which can therefore be an interesting functional additive in the food industry. The attempt to use them to enrich classic wheat bread shows that the obtained bread was characterized by interesting physicochemical and antioxidant properties. It was found that the bread with the 2% WF addition was characterized by the highest bread productivity and the lowest baking loss. In turn, the replacement of part of the flour with WF resulted in a reduction in the loaves’ volume and an increase in the crumb hardness, the greater their share in the recipe. Both the crust and the crumb were darker than the control bread. Increased redness of the bread crumb was also observed as the proportion of WF increased. Nevertheless, enriching the bread with walnut flowers had a positive effect on its antioxidant potential and the total polyphenol content. The values of these parameters, which indicate pro-health properties, increased statistically with the increasing addition of this plant supplement. The developed product, i.e., wheat bread enriched with walnut male flowers, can be recommended for consumers looking for food with an increased antioxidant potential and interesting sensory features due to the addition of unusual plant raw materials. The management of other by-products accompanying the cultivation of walnuts may contribute to the development of new food products with beneficial pro-health values. However, this issue requires further research. Nevertheless, the presented research results contribute by expanding the knowledge regarding the enrichment of wheat bread with unconventional plant additives, as well as by modifying existing recipes of fortified bread. Nowadays, the task of food technologists is to search for new functional additives that will contribute to the production of health-promoting food.

## Figures and Tables

**Table 1:** The parameters of laboratory baking process of wheat bread enriched with *J. regia* male flowers.

Flowers’ Share (*w*/*w* Flour Basis)	Dough Yield	Oven Loss	Total Baking Loss	Bread Yield
%	%	%	%	%
0.0 (control)	160.44 ± 1.50 ^a^	11.13 ± 1.08 ^b^	16.11 ± 0.77 ^ab^	134.59 ± 1.24 ^c^
0.5 (A)	159.70 ± 1.10 ^a^	11.51 ± 1.18 ^b^	15.40 ± 0.67 ^bc^	135.10 ± 1.07 ^c^
1.0 (B)	160.30 ± 2.00 ^a^	14.13 ± 0.49 ^a^	16.94 ± 0.37 ^a^	133.15 ± 0.59 ^c^
1.5 (C)	160.64 ± 1.80 ^a^	11.09 ± 0.82 ^b^	14.27 ± 0.32 ^cd^	137.72 ± 0.51 ^b^
2.0 (D)	161.21 ± 1.60 ^a^	8.68 ± 0.92 ^c^	12.32 ± 0.65 ^e^	141.35 ± 1.04 ^a^
2.5 (E)	163.41 ± 2.10 ^a^	10.72 ± 0.69 ^bc^	13.86 ± 0.46 ^d^	140.76 ± 0.75 ^a^

The means marked in the columns with the same letters do not differ at the significance level *p* ≤ 0.05.

**Table 2:** The quality parameters of wheat dough enriched with *J. regia* male flowers.

Flowers’ Share (*w*/*w* Flour Basis)	Volume of Bread	Specific Volume	Crumb Porosity Index
%	cm^3^	cm^3^/g	
0.0 (control)	675.00 ± 35.36 ^a^	3.21 ± 0.14 ^a^	70
0.5 (A)	650.00 ± 28.28 ^a^	3.07 ± 0.16 ^a^	50
1.0 (B)	650.00 ± 0.00 ^a^	3.12 ± 0.02 ^a^	60
1.5 (C)	600.00 ± 0.00 ^b^	2.79 ± 0.22 ^b^	70
2.0 (D)	590.00 ± 14.14 ^b^	2.69 ± 0.08 ^b^	70
2.5 (E)	565.00 ± 7.07 ^b^	2.62 ± 0.02 ^b^	80

The means marked in the columns with the same letters do not differ at the significance level *p* ≤ 0.05.

**Table 3:** Color of wheat dough enriched with *J. regia* male flowers.

Flowers’ Share (*w/w* Flour Basis)	L*	a*	b*	∆E
0.0 (control)	78.07 ± 0.20 ^a^	1.76 ± 0.02 ^b^	16.37 ± 0.07 ^c^	-
0.5	69.35 ± 0.16 ^b^	1.10 ± 0.18 ^d^	17.32 ± 0.40 ^b^	8.80 ± 0.24 ^e^
1.0	65.77 ± 0.24 ^c^	1.12 ± 0.08 ^d^	17.25 ± 0.20 ^b^	12.35 ± 0.11 ^d^
1.5	61.90 ± 1.22 ^d^	1.36 ± 0.26 ^cd^	18.25 ± 0.47 ^a^	16.29 ± 1.08 ^c^
2.0	59.20 ± 1.10 ^e^	1.47 ± 0.09 ^c^	17.44 ± 0.34 ^b^	18.91 ± 1.26 ^b^
2.5	55.19 ± 0.33 ^f^	2.26 ± 0.09 ^a^	18.37 ± 0.26 ^a^	22.97 ± 0.17 ^a^

The means marked in the columns with the same letters do not differ at the significance level *p* ≤ 0.05.

**Table 4:** Color of wheat bread enriched with *J. regia* male flowers.

Flowers’ Share (*w*/*w* Flour Basis)	L*	a*	b*	∆E
%		crust		
0.0 (control)	63.46 ± 4.60 ^a^	11.26 ± 2.58 ^a^	29.11 ± 1.18 ^a^	-
0.5	59.95 ± 1.18 ^a^	7.48 ± 0.58 ^b^	24.18 ± 1.85 ^b^	7.97 ± 1.56 ^b^
1.0	52.61 ± 2.57 ^c^	8.31 ± 1.46 ^b^	21.74 ± 1.51 ^b^	14.79 ± 3.86 ^a^
1.5	50.96 ± 2.88 ^c^	4.59 ± 0.35 ^c^	17.60 ± 0.21 ^c^	18.94 ± 2.68 ^a^
2.0	58.20 ± 2.59 ^ab^	3.96 ± 0.31 ^c^	15.99 ± 1.62 ^c^	17.17 ± 1.33 ^a^
2.5	53.87 ± 2.90 ^bc^	4.70 ± 0.98 ^c^	17.66 ± 1.56 ^c^	16.44 ± 0.63 ^a^
		crumb		
0.0 (control)	70.02 ± 0.28 ^a^	1.61 ± 0.09 ^e^	20.68 ± 0.36 ^a^	-
0.5	54.74 ± 0.74 ^b^	2.54 ± 0.10 ^d^	14.44 ± 0.23 ^c^	16.53 ± 0.63 ^d^
1.0	46.01 ± 0.82 ^c^	3.53 ± 0.14 ^c^	15.09 ± 0.43 ^b^	24.72 ± 0.88 ^c^
1.5	40.33 ± 0.66 ^e^	4.21 ± 0.21 ^a^	14.70 ± 0.51 ^bc^	30.40 ± 0.41 ^a^
2.0	42.61 ± 0.27 ^d^	3.86 ± 0.11 ^b^	14.16 ± 0.08 ^c^	28.27 ± 0.24 ^b^
2.5	39.94 ± 0.07 ^e^	4.24 ± 0.13 ^a^	14.33 ± 0.17 ^c^	30.85 ± 0.26 ^a^

The means marked in the columns with the same letters do not differ at the significance level *p* ≤ 0.05.

**Table 5:** Textural parameters of wheat bread enriched with *J. regia* male flowers.

Flowers’ Share (*w*/*w* Flour Basis)	Hardness	Elasticity	Cohesiveness	Gumminess	Chewiness	Resilience
%	(N)	(-)	(-)	(N)	(N)	(-)
0.0 (control)	13.92 ± 2.03 ^b^	0.77 ± 0.06 ^a^	0.63 ± 0.06 ^ab^	8.81 ± 1.65 ^b^	6.79 ± 1.53 ^ab^	0.31 ± 0.03 ^ab^
0.5	15.52 ± 1.43 ^b^	0.76 ± 0.01 ^a^	0.64 ± 0.04 ^ab^	9.92 ± 1.03 ^b^	7.52 ± 0.74 ^a^	0.31 ± 0.03 ^ab^
1.0	13.72 ± 1.76 ^b^	0.75 ± 0.02 ^a^	0.67 ± 0.05 ^a^	9.24 ± 1.71 ^b^	6.94 ± 1.36 ^a^	0.33 ± 0.04 ^a^
1.5	15.63 ± 0.61 ^b^	0.57 ± 0.05 ^c^	0.58 ± 0.03 ^b^	9.06 ± 0.30 ^b^	5.20 ± 0.48 ^b^	0.25 ± 0.03 ^c^
2.0	14.49 ± 0.45 ^b^	0.67 ± 0.04 ^b^	0.61 ± 0.03 ^ab^	8.81 ± 0.14 ^b^	5.91 ± 0.30 ^ab^	0.28 ± 0.01 ^bc^
2.5	21.02 ± 2.51 ^a^	0.59 ± 0.03 ^c^	0.59 ± 0.02 ^b^	12.34 ± 1.63 ^a^	7.33 ± 1.23 ^a^	0.26 ± 0.02 ^bc^

The means marked in the columns with the same letters do not differ at the significance level *p* ≤ 0.05.

## Data Availability

Not applicable.
